# Pyruvate‐lactate exchange and glucose uptake in human prostate cancer cell models. A study in xenografts and suspensions by hyperpolarized [1‐^13^C]pyruvate MRS and [^18^F]FDG‐PET

**DOI:** 10.1002/nbm.4362

**Published:** 2020-07-14

**Authors:** Frits H.A. van Heijster, Sandra Heskamp, Vincent Breukels, Andor Veltien, Gerben M. Franssen, Kees (C).F.J. Jansen, Otto C. Boerman, Jack A. Schalken, Tom W.J. Scheenen, Arend Heerschap

**Affiliations:** ^1^ Department of Radiology and Nuclear Medicine Radboud University Medical Center Nijmegen The Netherlands; ^2^ Department of Urology Radboud University Medical Center Nijmegen The Netherlands

**Keywords:** ^13^C MRS, ^18^F[FDG], hyperpolarization, PET, prostate cells, pyruvate, xenografts

## Abstract

Reprogramming of energy metabolism in the development of prostate cancer can be exploited for a better diagnosis and treatment of the disease. The goal of this study was to determine whether differences in glucose and pyruvate metabolism of human prostate cancer cells with dissimilar aggressivenesses can be detected using hyperpolarized [1‐^13^C]pyruvate MRS and [^18^F]FDG‐PET imaging, and to evaluate whether these measures correlate. For this purpose, we compared murine xenografts of human prostate cancer LNCaP cells with those of more aggressive PC3 cells. [1‐^13^C]pyruvate was hyperpolarized by dissolution dynamic nuclear polarization (dDNP) and [1‐^13^C]pyruvate to lactate conversion was followed by ^13^C MRS. Subsequently [^18^F]FDG uptake was investigated by static and dynamic PET measurements.

Standard uptake values (SUVs) for [^18^F]FDG were significantly higher for xenografts of PC3 compared with those of LNCaP. However, we did not observe a difference in the average apparent rate constant *k*_pl_ of ^13^C label exchange from pyruvate to lactate between the tumor variants. A significant negative correlation was found between SUVs from [^18^F]FDG PET measurements and *k*_pl_ values for the xenografts of both tumor types. The *k*_pl_ rate constant may be influenced by various factors, and studies with a range of prostate cancer cells in suspension suggest that LDH inhibition by pyruvate may be one of these. Our results indicate that glucose and pyruvate metabolism in the prostate cancer cell models differs from that in other tumor models and that [^18^F]FDG‐PET can serve as a valuable complementary tool in dDNP studies of aggressive prostate cancer with [1‐^13^C]pyruvate.

## Graphical abstract

Differences in energy metabolism in murine xenografts of prostate cancer are found using hyperpolarized MR and PET. A significant negative correlation was seen between PET‐derived SUVs and ^13^C‐MR‐derived *k*_pl_ values for the xenografts of both tumor types. Standard uptake values (SUV) for [^18^F]FDG were significantly higher for PC3 compared to LNCaP. Energy metabolism in prostate cancer cell models differ from other tumor models and [^18^F]FDG‐PET can serve as a complementary tool in dDNP studies of aggressive prostate cancer with [1‐^13^C]pyruvate.

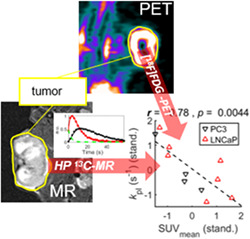

## 1 INTRODUCTION

Prostate cancer is responsible for one in 15 cancer related deaths in men worldwide.^1^ Distinguishing between aggressive and non‐aggressive tumors is a challenge, and accurate characterization can avoid unnecessary treatments.^2^, ^3^ One of the hallmarks of cancer is deregulated energy metabolism into a more glycolytic phenotype even in the presence of oxygen, the so‐called Warburg effect.^4^ Therefore, malignant cells often show high rates of aerobic glycolysis,^5^, ^6^, ^7^ with high uptake of glucose and excretion of large amounts of lactate, an end product of (aerobic) glycolysis.

**FIGURE 1:**
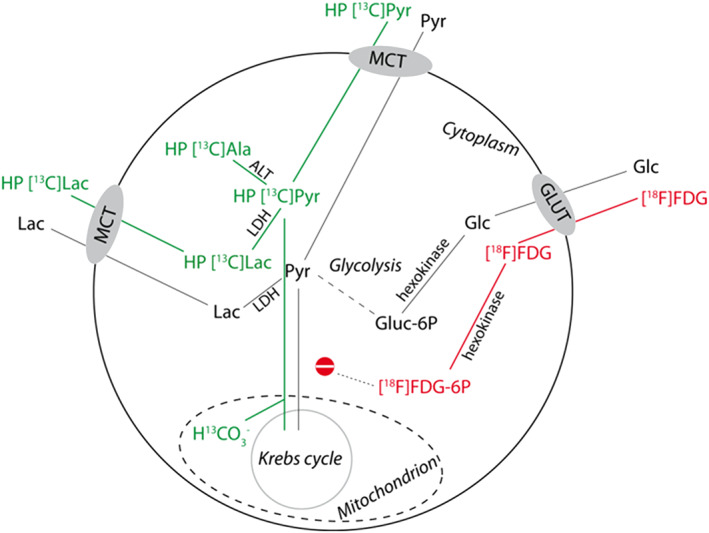
Metabolism involved in energy production in tumor cells and pathways involved in [1‐^13^C]pyruvate and [^18^F]FDG metabolism. Malignant cells often have high energy demands reflected in high uptake of glucose (Glc) by GLUT transporters and high conversion rates of pyruvate (Pyr) into lactate (Lac) by LDH (Warburg effect). Hyperpolarized (HP) [1‐^13^C]pyruvate can enter the cells via MCT transporters and is rapidly converted to HP [1‐^13^C]lactate, which is excreted (green pathway). Using radiolabeled [^18^F]FDG, a glucose analog, the uptake and subsequent phosphorylation of glucose can be estimated (red pathway)

Cancer metabolism can be investigated non‐invasively by dynamic carbon‐13 (^13^C) MRI and MRS using ^13^C‐labeled metabolic substrates, and by positron emission tomography (PET) using radiolabeled 2‐deoxy‐2‐[^18^F]fluoro‐d‐glucose ([^18^F]FDG) (Figure 1). Conventional in vivo ^13^C MRS suffers from a low signal‐to‐noise ratio (SNR) due to a low gyromagnetic ratio.^8^ Enhancing signal strength by hyperpolarization using dissolution dynamic nuclear polarization (dDNP) increases the SNR by a factor greater than 10^4^, allowing single‐shot detection of metabolites in vivo.^9^, ^10^ After injecting a hyperpolarized ^13^C‐labeled substrate, its signal and that of its metabolic products can be followed within a time window of the order of the *T*_1_ of the excited spins. A common substrate used in these experiments is [1‐^13^C]pyruvate because of its relatively long *T*_1_ relaxation time (~40 s), fast metabolism and safe application.^11^ The role of pyruvate in aerobic glycolysis and redox equilibrium makes it a highly relevant substrate to study these key elements of energy metabolism in malignant cells. Hyperpolarization using dDNP has been used extensively in the last two decades to monitor the in vivo exchange of pyruvate to lactate by lactate dehydrogenase (LDH) and of pyruvate to alanine by alanine transaminase (Figure 1).^12^, ^13^ Malignant tissue often shows higher lactate and alanine signals than healthy tissue after injection of hyperpolarized [1‐^13^C]pyruvate, reflecting product accumulation in the end steps of glycolysis.^14^, ^15^, ^16^, ^17^ Changes in rates of pyruvate metabolism can be used to monitor treatment response.^18^

The higher glucose uptake in energy demanding malignant tissue is widely employed in PET imaging of tumors using the glucose analog [^18^F]FDG.^19^ Upon intravenous injection, [^18^F]FDG is taken up by cancer cells mediated by glucose transporters (GLUTs) and is subsequently phosphorylated to [^18^F]FDG‐6‐phosphate, which is trapped in the cell and not metabolized further. Thus [^18^F]FDG uptake provides an estimate of glucose uptake and its phosphorylation in the first step of glycolysis (Figure 1).^20^ In prostate cancer [^18^F]FDG uptake is increased in aggressive tumor lesions with a high Gleason score.^21^, ^22^

The human prostate cancer lymph node metastasis cell line LNCaP is used in studies to represent early stage (metastatic) prostate cancer. LNCaP is androgen responsive, produces prostate specific antigen (PSA) and is one of the more differentiated human prostate cancer cell lines.^23^, ^24^ The human prostate cancer bone metastasis cell line PC3, on the other hand, does not express RNA for PSA or the androgen receptor and is considered to represent castration resistant late stage disease, with a more aggressive phenotype.^24^, ^25^

The aim of this study is to investigate whether subcutaneous xenografts of LNCaP and PC3, as models of early and late stage prostate cancer, can be distinguished from each other by the dDNP hyperpolarized [1‐^13^C]pyruvate to [1‐^13^C]lactate exchange rate *k*_pl_, measured by ^13^C MRS, and by the standardized uptake value (SUV) of [^18^F]FDG measured by PET. Furthermore, we investigated whether the outcomes of these two parameters are correlated and may have complementary roles in studies of prostate cancer models.

As the exchange rate constant *k*_pl_ strongly depends on the local pyruvate concentration,^26^, ^27^ we also determined its dependence on a range of pyruvate concentrations in suspensions of LNCaP and PC3 cells and compared these with other prostate cells and with EL4 lymphoma cells, for which the effect of pyruvate concentration on ^13^C label exchange has been previously investigated.^28^

## **2** MATERIALS AND METHODS

### 2.1 Animal models

**FIGURE 2:**
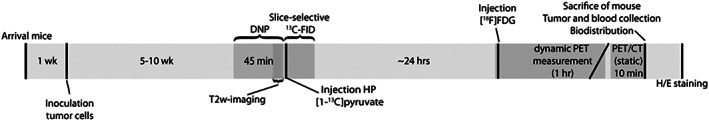
Time line of mouse experiments. After the arrival of the male Balb/c nude mice LNCaP or PC3 cells were inoculated and tumors allowed to grow for 5‐10 weeks. When the tumors had reached an appropriate size to be measured, first MR experiments were performed. [1‐^13^C]pyruvate was hyperpolarized (dDNP) and after *T*_2_‐weighted images were acquired it was injected intravenously and slice selective ^13^C‐FIDs were obtained under anesthesia. About 24 h later the mice were injected with [^18^F]FDG and static or dynamic PET measurements were performed. After sacrificing the animals, tumors and blood were collected and biodistributions were determined. Finally, tissue coupes were made of the tumors and H/E stainings were performed

LNCaP and PC3 tumor cells were grown in RPMI‐1640 medium (Gibco, Life Technologies, Bleiswijk, Netherlands) supplemented with fetal calf serum (10%) and 2 mM glutamine (Gibco, Life Technologies). In total, 30 nude mice (10 weeks old, male, BALB/cAnNRj‐Foxn1nu/Foxn1nu, Janvier) were used for the experiments, split into three groups of 10 animals, measured at three different timepoints. All animal experiments were conducted according to institutional guidelines and regulations and were approved by the national Central Animal Experiments Committee (CCD) and the local animal welfare body (RU‐DEC 2015‐0071). Five randomly selected mice per group were subcutaneously injected with 3 × 10^6^ LNCaP cells (in matrigel: RPMI‐1640 2:1) and five mice with 3 × 10^6^ PC3 cells (in matrigel: RPMI‐1640 1:2) in the right hind leg. The mice with PC3 and LNCaP tumors were housed in two separate cages. When the tumors reached a size of 0.25‐0.50 cm^3^, hyperpolarized [1‐^13^C]pyruvate MR experiments were done. Subsequently, [^18^F]FDG‐PET was performed 24 h later to allow the mice to recover from the anesthesia during the MR experiments and from the injected pyruvate bolus (Figure 2).

### **2.2** Hyperpolarization

[1‐^13^C]pyruvate (2 M) was dissolved in a D_2_O:ethanol‐d6 mixture (2:1) and TEMPOL (4‐hydroxy‐TEMPO) radical (30 mM) was added. In 20 cases ^13^C‐urea was dissolved in a glycerol:H_2_O:D_2_O mixture (6:1:3) and co‐polarized to check the arrival of the hyperpolarized material at the tumor. The sample was hyperpolarized at about 1.15 K by irradiation using a microwave source (95 GHz) in a home‐built 3.35 T DNP polarizer.^29^ When polarization levels reached a plateau (after ~45 min) the sample was quickly removed from the DNP machine by dissolution in hot buffer (4 mL, phosphate buffered saline, pH 7.5, 180 °C, 12 bar), guiding the solution (final concentration 80 mM) to the MRI scanner via a capillary in 4‐5 s before it was injected into the tail vein of the mouse.

### **2.3** MRI and MRS

**FIGURE 3:**
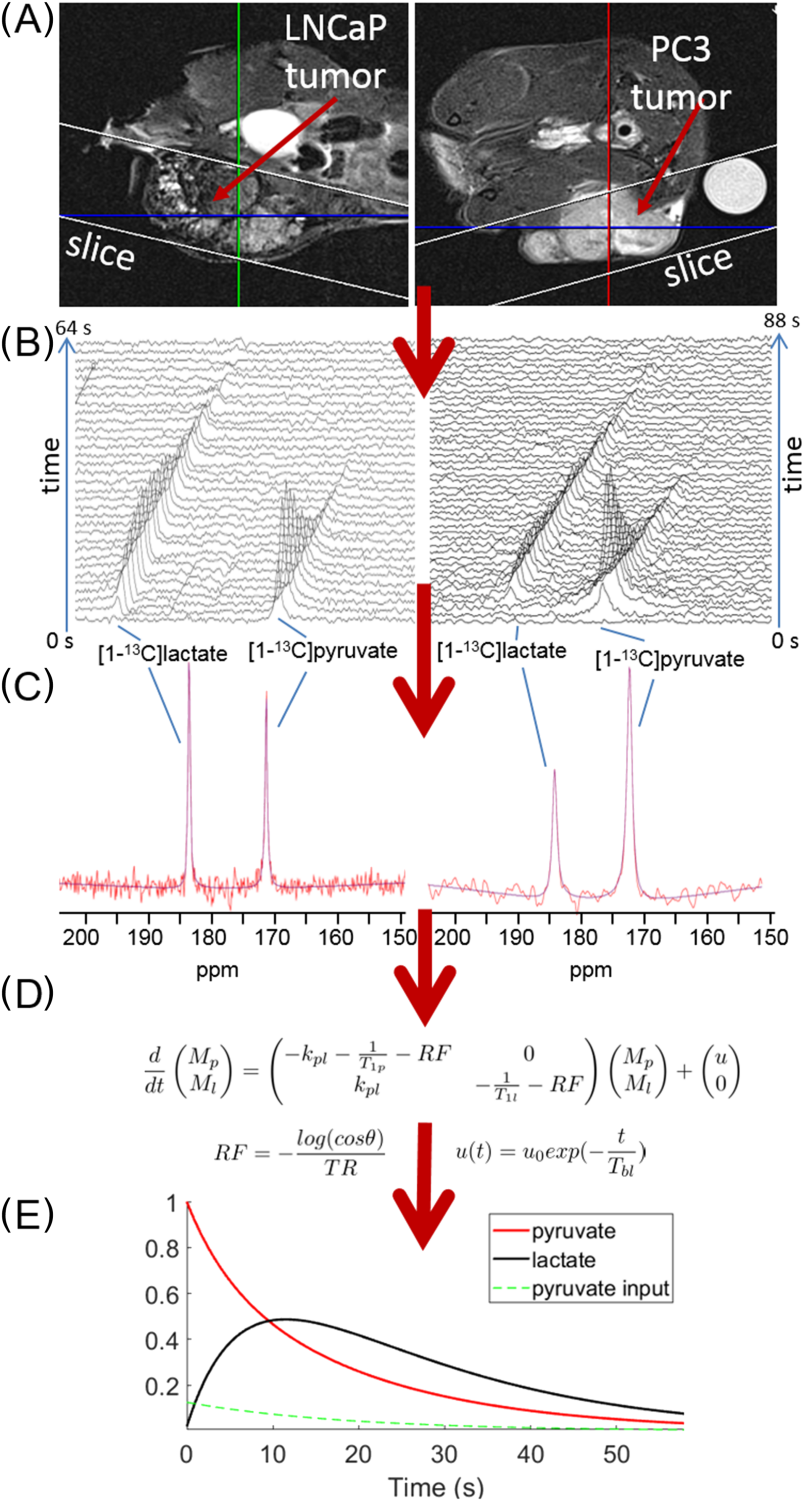
Acquisition and analysis of data in hyperpolarization experiments. A, *T*_2_‐weighted MR images of mice bearing LNCaP (left) or PC3 (right) tumor. The selected slice is indicated by white lines. After injection of HP [1‐^13^C]pyruvate a series of slice‐selective ^13^C‐FIDs is recorded (*T*_R_ = 2 s, *θ* = 30°). B, Signals of hyperpolarized [1‐^13^C]pyruvate and lactate are detected over time. C, The signals of [1‐^13^C]pyruvate and [1‐^13^C]lactate are fitted using the AMARES fitting routine in jMRUI. D, Kinetic model used to fit time courses of pyruvate and lactate amplitudes (*M*_p_ and *M*_l_). The reaction rate *k*_pl_ is derived from this model. Variables included a PIF *u*(*t*) and the flip angle *θ*. The reverse reaction rate is assumed to be zero (*k*_lp_ = 0) and *T*_1p_ = 40 s, *T*_1l_ = 35 s. E, Time courses of relative signal integrals of pyruvate and lactate using a pyruvate input finction (PIF)

Mice were anesthetized with 1.5‐2.5% isoflurane in a mixture of medical air and oxygen (2:1). A catheter was inserted into the tail vein. Body temperature was monitored with a rectal thermometer and maintained using heated air. Mice were placed prone in the magnet of a preclinical 7 T MR system (ClinScan, Bruker Biospin GmbH, Rheinstetten, Germany) with a dedicated home‐built probe (^13^C surface coil *d* = ~1.5 cm, ^1^H birdcage coil *d* = ~10 cm). After localization of the tumors with fast gradient echo imaging, *T*_2_‐weighted images were acquired (coronal, transversal and axial) using a turbo‐spin‐echo sequence (*T*_E_ = 38 ms, *T*_R_ = 3 s, 256 × 256 matrix, 40 mm × 40 mm, 20 slices of 1 mm thickness) to determine the morphology of the tumors. Subsequently, hyperpolarized [1‐^13^C]pyruvate (300 μL, 80 mM) was injected intravenously. The conversion of [1‐^13^C]pyruvate into [1‐^13^C]lactate was followed by measuring a slice‐selective ^13^C‐FID (free induction decay) every 2 s (flip angle, *θ* = 30°, bandwidth = 20 kHz) with the slice placed such that it contained mainly tumor, and as little as possible other tissue (Figures 3A and 3B).

To determine how much ^13^C label exchange between pyruvate and lactate is to be expected in tumor‐free muscle tissue, one mouse without tumor was measured and analyzed in a similar manner to the mice with tumors, placing the slice for the hyperpolarization experiment at the right hind leg of the mouse, covering mostly muscle tissue.

### **2.4** Kinetic modeling of hyperpolarization data acquired from tumors in mice

The time series of ^13^C FIDs were fitted using the AMARES algorithm in jMRUI software,^30^, ^31^, ^32^ applying the same prior knowledge (soft constraints on linewidth and frequency) for the pyruvate and lactate signals at every time point for each mouse (Figure 3C). Different constraints on linewidth were used for each mouse due to differences in shimming and heterogeneity of the tumor. When the SNR of the measurement was too low for reasonable analysis, the data was excluded from the experiment. Time courses of pyruvate and lactate signal integrals were fitted using ordinary differential equation solvers (MATLAB, MathWorks) by a one‐pool unidirectional kinetic model^14^, ^33^ including a modified input function *u*(*t*) = *u*_0_ exp{−*t*/*T*_bl_} (Figure 3D and 3E). We set the longitudinal relaxation rates for pyruvate and lactate to be *T*_1p_ = 40 s and *T*_1l_ = 35 s and fitted the [1‐^13^C]pyruvate to [1‐^13^C]lactate exchange rate constant *k*_pl_, the flip angle *θ* responsible for the RF decay term in the model and both the amplitude *u*_0_ and decay time *T*_bl_ of the input function. As well as fitting the time series data to a kinetic model we also used a model‐free approach^34^ by first determining the area under the curve (AUC) of the changing pyruvate and lactate signal intensities, and second calculating the ratio between these two AUCs. We did this for AUC ratios of lactate over pyruvate both for the fitted signals (by calculating the AUCs fitted by the kinetic model) and using the raw AMARES amplitudes (by calculating the Riemann sum). Both AUC ratios were calculated over the same time period as the kinetic fits.

### **2.5** Static PET imaging

One day after the in vivo MR measurements, a group of nine mice (PC3 *N* = 5, LNCaP *N* = 4) were anesthetized using isoflurane/O_2_ and intravenously injected with [^18^F]FDG (0.2 mL, 6.9 ± 1.5 MBq). Mice were maintained at 37 °C and kept under anesthesia for 1 h, followed by PET/CT (computed tomography) imaging (Inveon preclinical PET/CT scanner, Siemens Healthcare Molecular Imaging). Static PET scans were acquired for 15 min (Figure 2A) followed by CT (80 kV, 500 μA, 300 ms exposure time) for anatomic localization. Reconstructions were performed using Inveon Acquisition Workplace software, version 2.04 (Siemens Preclinical Solutions), using the OSEM3D/SPMAP algorithm for PET (matrix size 256 × 256) and the modified Feldkamp algorithm for CT (Shepp‐Logan filter).

### **2.6** Dynamic PET imaging

For dynamic PET imaging, 16 mice were injected 24 h after the MR measurement with 0.2 mL [^18^F]FDG. One group of 10 mice (PC3 *N* = 5, LNCaP *N* = 5) received 13.3 ± 2.0 MBq and another of 6 mice (PC3 *N* = 4, LNCaP *N* = 2) received 8.4 ± 1.7 MBq. Mice were kept under anesthesia and maintained at 37 °C for 10 min before intravenous injections via a tail vein canula whilst inside the PET imager. Transmission scans were acquired for 5 min and subsequently [^18^F]FDG uptake was measured dynamically for 65 min. Dynamic PET frames were reconstructed using OSEM3D/SPMAP (matrix size 256 × 256) for time frames of 3 × 10s, 18 × 5 s, 13 × 1 min and 5 × 10 min, and static PET images after 60 min were reconstructed for all mice.

### **2.7** PET data ROI analysis

After reconstruction, regions of interest (ROIs) were drawn around the tumors using Inveon Research Workplace (IRW) and the [^18^F]FDG avid tissue was selected using a cut‐off value of 30% of the hottest voxel in the ROI. Standardized uptake values (SUV_max_, hottest voxel; SUV_mean_, mean over tumor tissue) were calculated from the reconstructed static PET images (60 min after injection) of all three groups. An ROI was placed inside the aorta of the mouse to estimate a blood input function for the radiotracer.

### **2.8** ex vivo biodistribution and H/E stains

After the PET measurements, mice were euthanized and blood and tumor were collected to determine [^18^F]FDG uptake. Tumor tissue was formalin‐fixed and embedded in paraffin. Tumor sections were stained with hematoxylin and eosin (H/E) to determine the percentage of viable tumor tissue and to determine the presence of necrosis and hemorrhages (Figure 2A).

### **2.9** Kinetic modeling of dynamic PET data

The PET frames reconstructed from the dynamic PET measurements of the tumor ROIs were analyzed, using IRW software. Two‐tissue‐compartment models (2TCMs), which take into account [^18^F]FDG uptake from blood (*k*_1_) and [^18^F]FDG‐6‐phosphate formation (*k*_3_) and their reverse reaction rates and *k*_2_ and *k*_4_, were fitted, and Patlak analysis was performed. Both the irreversible Sokoloff 3K (*k*_4_ = 0) and reversible Phelps 4K (*k*_4_ ≠ 0) 2TCM models were fitted to the data.^35^, ^36^ The fraction of blood inside the tumor, *V*_b_, was set to 0 and the injection time delay *t*_d_ was set to a value depending on the aorta input curves, between −10 and −30 s. Then, the [^18^F]FDG uptake constants *K*_i_ (=*K*_1_ *k*_3_ / *k*_2_+*k*_3_) and volumes of radiotracer distribution *V*_D_ (=*K*_1_ *k*_3_ / *k*_2_ *k*_4_) and the non‐displaceable fraction of radiotracer distribution *V*_DND_ (=*K*_1_/*k*_2_) were determined for both models. In addition to this, *K*_i_ was determined from the Patlak curves.

### **2.10** Correlation between pyruvate to lactate exchange and FDG uptake

To find a possible correlation between pyruvate to lactate exchange and FDG uptake, Pearson's *r* correlations were calculated for each tumor group and for combined groups. We investigated whether fitted values for pyruvate to lactate exchange rate (*k*_pl_) or pyruvate to lactate ratios ([lac/pyr]) correlated with standard uptake values (SUV) and the [^18^F]FDG uptake parameter (*K*_i_). To determine whether there is a more general correlation between these measures both the *k*_pl_ and SUV values were standardized before combining the datasets. This was done by first calculating the mean and standard deviation for all successful hyperpolarization experiments and for all successful PET experiments separately. Subsequently the datasets were normalized (*μ* = 0, *σ* = 1) to calculate *z*‐scores and the correlations were calculated.

To provide a measure of the ratio between lactate production and glucose uptake we also calculated the ratios of *k*_pl_ (and AUC ratios [lac/pyr]_fit_ and [lac/pyr]_raw_) over SUV_max_ and SUV_mean_.

### **2.11** Hyperpolarization studies of prostate cancer cells in suspension

Prostate cancer metastasis cell lines LNCaP, PC3 and DU‐145, prostate cell line EP156^37^ and murine lymphoma cell line EL4, used in this study, were authenticated with PowerPlex 21 STR profiling (Eurofins Genomics, Ebersberg, Germany). Culture conditions and measurement of the [1‐^13^C]pyruvate to lactate exchange rate in suspensions of these cells inside a 5 mm NMR tube have been described previously.^29^ In short, the tumor cells were grown in 25 cm^2^ cell culture flasks in standard medium. Subsequently, they were dissolved in fresh medium and transferred to a 5 mm NMR tube (Shigemi Inc. Allison Park, PA, USA), which was placed in the magnet of an Avance 500 MHz spectrometer (Bruker Biospin GmbH, Rheinstetten, Germany). Hyperpolarized [1‐^13^C]pyruvate together with unlabeled lactate was then injected into the NMR tube with cells to reach the desired pyruvate concentrations (ranging from 2.5 mM to 15 mM) and 15 mM lactate (present in the dissolution buffer) in the solution, which was kept at 37 °C. Starting just before hyperpolarized pyruvate injection, ^13^C MR spectra, from which the signal integrals for hyperpolarized [1‐^13^C]pyruvate and [1‐^13^C]lactate were determined as a function of time, were continuously recorded. The pyruvate to lactate exchange rate constant *k*_pl_ (1/s/million cells) was calculated by fitting these time curves to a one‐directional kinetic model as described before.^29^ Finally, the *k*_pl_ values were multiplied by the initial hyperpolarized [1‐^13^C]pyruvate concentration and the sample volume, and divided by the number of cells, to obtain ^13^C exchange flux values (nmol/s/million cells). Per cell type these flux values (*V*_obs_) were fitted to the Michaelis‐Menten equation *V*_obs_ = (*V*_max_ + [pyr]) / *K*_m_[pyr] to obtain the maximum flux *V*_max_ and affinity constant *K*_m_.

## **3** RESULTS

### 3.1 MRI

**FIGURE 4:**
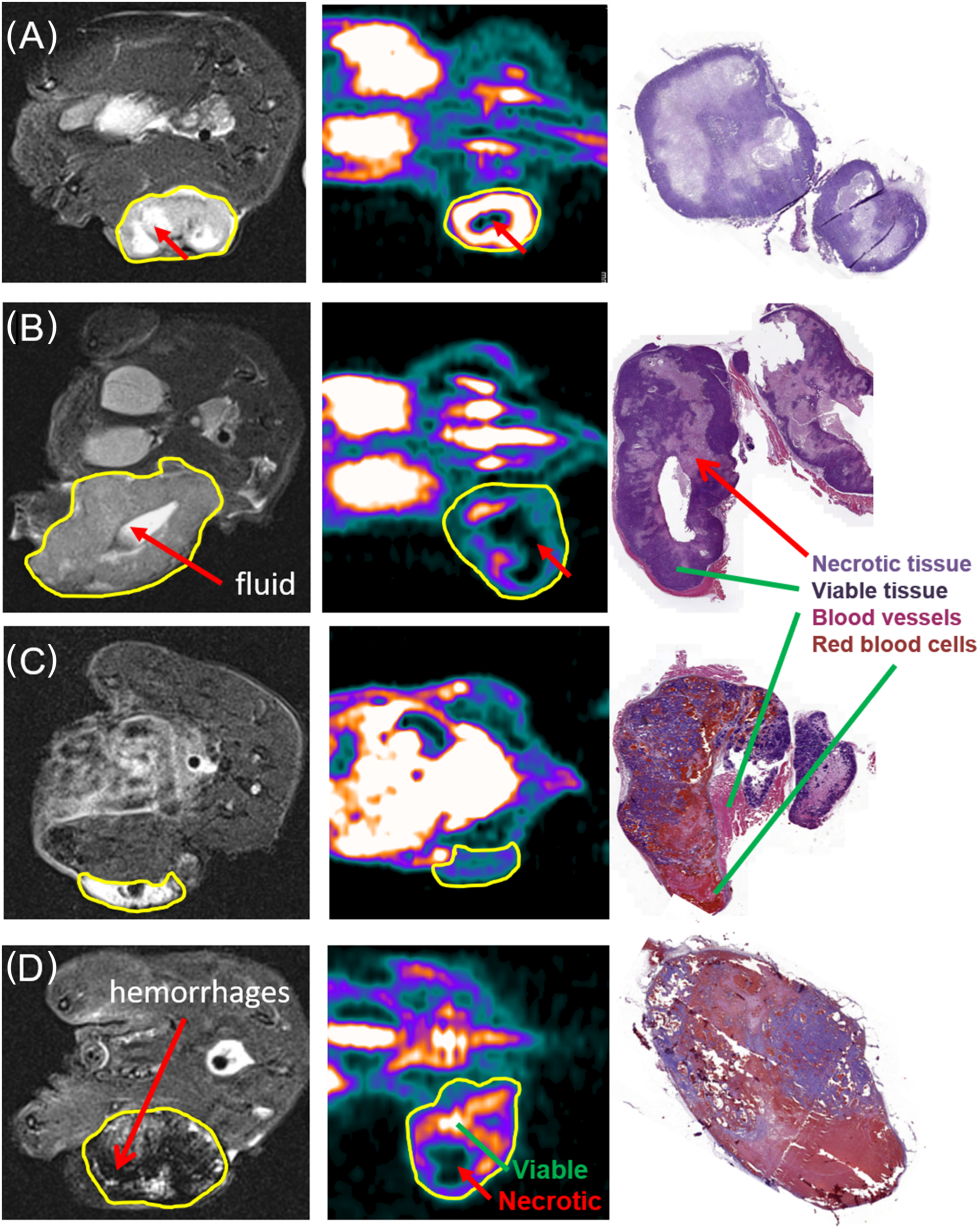
*T*_2_‐weighted MR, [^18^F]FDG PET images and H/E stainings for PC3 and LNCaP tumors. Left‐hand column, transversal *T*_2_‐weighted MR images; middle column, corresponding [^18^F]FDG PET images; right‐hand column, corresponding H/E stains. A, B, PC3 tumors; C, D, LNCaP tumors. The tumor regions are indicated in yellow and the necrotic or non‐viable tissue is indicated by red arrows. The PC3 tumor in B shows an irregular shape and fluid inside, the LNCaP tumor in C is cigar shaped and located just below the skin, and the tumor in D is more spherical, containing many hemorrhages. The presence of necrosis and viable tissue is confirmed in the H/E stained coupes of the tumors in the right‐hand column

*T*_2_‐weighted anatomical MR images were acquired to visualize tumor morphology and to determine where to position the slice for the ^13^C FID acquisitions in the dDNP experiments (Figures 3 and 4). The *T*_2_‐weighted MR images revealed substantial variations in morphology for the different tumors (Figure 4). The PC3 tumors varied in shape from spherical (Figure 4A) to irregular (Figure 4B). Some had a necrotic core (Figure 4A) or included cysts with fluid inside (Figure 4B). LNCaP tumors mostly developed in an ellipsoid shape under the skin (Figure 4C), but some were larger and contained hemorrhages (Figure 4D). The presence of these tumor features was validated by comparing MR images with the corresponding [^18^F]FDG uptake images and with the histopathology of the tumors described below, where the absence of [^18^F]FDG uptake in the center of the tumors suggests necrosis or hemorrhage (Figure 4).

### **3.2** Hyperpolarized ^13^C pyruvate conversions in tumor xenografts monitored by ^13^C MRS

Immediately after injection of hyperpolarized [1‐^13^C]pyruvate in the tail vein of the mouse, the ^13^C MR spectrum of the selected slice showed a high signal intensity at 171 ppm (parts per million) for pyruvate (Figure 3B and 3C), which further increased in subsequent spectra as pyruvate continued to enter the tumor‐containing slice. After about three spectra the pyruvate signal decreased due to longitudinal relaxation and fast conversion to lactate as seen by the appearance of a signal for lactate at 183 ppm very shortly after the first pyruvate signal was observed. This lactate signal first increased in subsequent measurements, and then declined. In total 200 FIDs were recorded, or fewer when the SNR became insufficient for further signal analysis (~40‐50 s after the first ^13^C signal was detected).

### **3.3** Kinetic modeling of ^13^C MR signals of hyperpolarized ^13^C labeled compounds in tumor tissue

**FIGURE 5:**
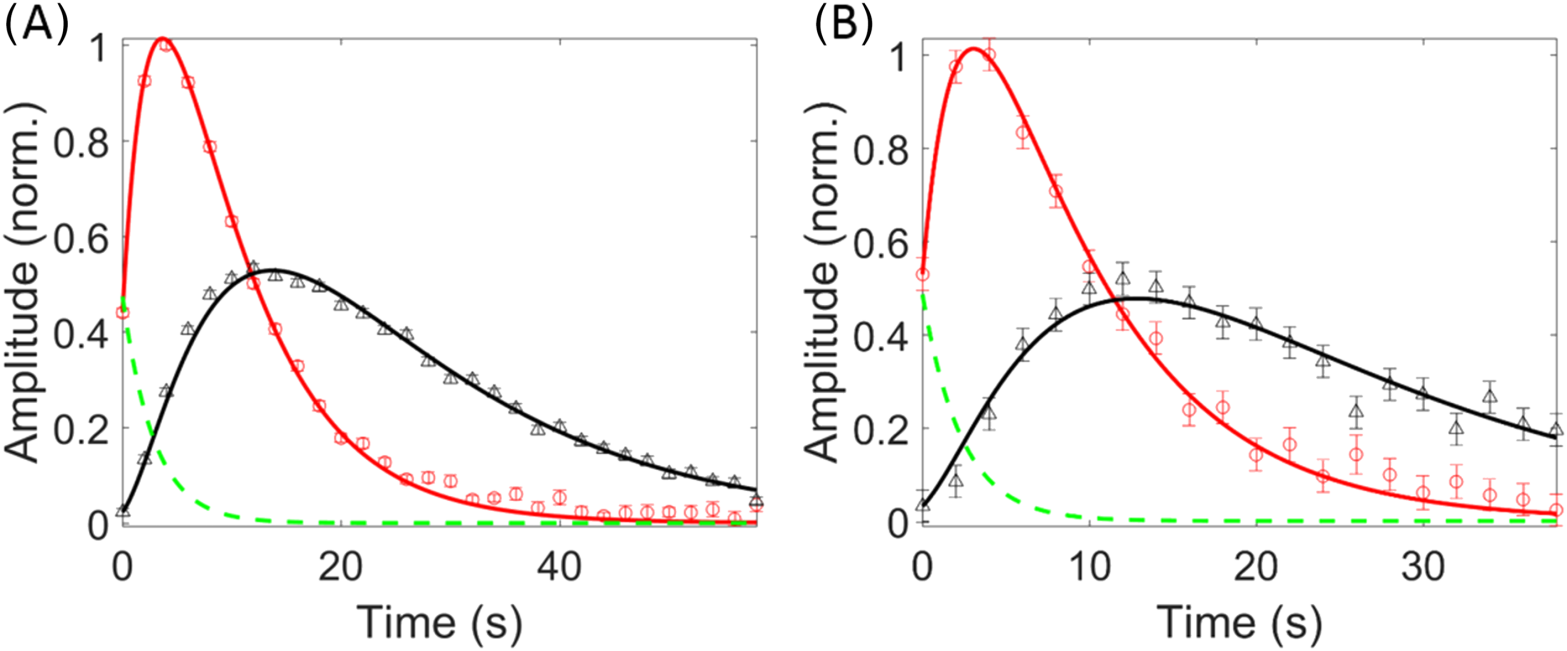
Fit of pyruvate and lactate ^13^C signal amplitudes as a function of time using the kinetic model: LNCaP (A) and PC3 (B) tumors. The amplitudes of pyruvate (red circles) and lactate (black triangles) were obtained by AMARES in jMRUI and their time variation fitted to the kinetic model presented in Figure 3D. Fitting variables: *k*_pl_, *u*_0_, *T*_bl_ and *θ*. The input function *u*(*t*) is shown in green. The first point of the fit was the first point at which a signal was detected with sufficient SNR. Fits with the flip angle fixed at *θ* = 30° are shown in Figure S1

To analyze the time courses of the ^13^C signals of pyruvate and lactate with a kinetic model, their signal integrals were determined by fitting the ^13^C signals of the whole time series using AMARES (Figure 3C). These time courses of the pyruvate and lactate signal integrals were fitted to a one‐pool unidirectional kinetic model (Figure 3D) and plotted for visualization (Figure 4). As not all pyruvate circulating in blood enters the tumor at once, we included in the model an input function *u*(*t*) = *u*_0_ exp{*t*/*T*_bl_}, which represents a constant inflow of hyperpolarized pyruvate during the time window of the experiment with a characteristic decay time *T*_bl_, taking into account that this pyruvate is diluted in blood, is taken up elsewhere in the body, and experiences different excitation pulse angles at different locations in the body and in the selected slice. Using this model, the label exchange rate constant *k*_pl_ was fitted as a measure of pyruvate to lactate conversion in addition to input function amplitude *u*_0_ and decay time *T*_bl_, since these can be different for each mouse and tumor. Either the very first time point showing a pyruvate signal or the time with maximum pyruvate signal was used as first time point for the fit. In addition to this, either pulse angle *θ* was kept at 30° (the angle intended to be applied in the experiments) or *θ* was fitted along with *k*_pl_, *u*_0_ and *T*_bl_. Including pulse angle *θ* in the model resulted in better fits than with the pulse angle fixed (Figure 5, Figure S1). If no pyruvate input function (PIF) was taken into account, the fits suffered from large residues and unrealistic values were obtained for the fitted parameters. Fitting with or without angle *θ*, the *k*_pl_ values for the LNCaP tumors (0.10 ± 0.06 or 0.12 ± 0.03/s) were not different from those of the PC3 tumors (Table 1).

**TABLE 1:** Pyruvate metabolic values obtiained from kinetic model fits of DNP time curves

	*k*_pl_ (1/s)				Lactate/pyruvate ratio (fitted data)				Lactate/pyruvate ratio (raw data)			
Fitted parameters	PC3	LNCaP	*p*	Muscle	PC3	LNCaP	*P*	Muscle	PC3	LNCaP	*P*	Muscle
*k*_pl_ *, u*_0_ *, T*_bl_ *,* pyr_max_	0.11 ± 0.03	0.12 ± 0.03	0.83	4 × 10^−7^	1.2 ± 0.3	1.3 ± 0.5	0.44	0.2	1.2 ± 0.3	1.3 ± 0.4	0.59	0.2
*k*_pl_ *, θ, u*_0_ *, T*_bl_ *,* pyr_max_	0.08 ± 0.03	0.10 ± 0.06	0.55	5 × 10^−3^	1.2 ± 0.4	1.4 ± 0.4	0.54	0.2	1.2 ± 0.3	1.3 ± 0.4	0.59	0.2

The fitted values of *k*_pl_ for the different fits per tumor are shown in the table along with the ratio of lactate/pyruvate AUCs (either integrated fits/AUC or sum of raw amplitudes). In addition to *k*_pl_ also input function parameters *u*_0_ and *T*_bl_ are fitted; the pulse angle *θ* was either set to 30° or fitted as well. Fitting started at the highest pyruvate intensity pyr_max_. Two‐sample *t*‐tests are performed to test for difference between PC3 and LNCaP; no difference is found. Negligible values for *k*_pl_ are found in the muscle of a mouse without tumor, measured to test if muscle tissue shows significant pyruvate conversion in the absence of tumor tissue.

The lactate over pyruvate AUC ratios were similar for LNCaP and PC3 tumors regardless of whether these ratios were calculated from the raw data or the fitted values (Table 1).

### **3.4** ^13^C MRS of muscle tissue in mouse as control

One mouse without tumor was measured in the same way as the tumor‐bearing mice to check whether muscle tissue shows a significant pyruvate to lactate conversion that could influence the experiments in tumor‐bearing mice. Fitting the limited number of pyruvate and lactate signals with a high enough SNR to the kinetic models resulted in negligible *k*_pl_ values (Table 1).

### **3.5** PET imaging

To estimate the relative uptake of glucose in the tumors, we administered [^18^F]FDG intravenously to the LNCaP and PC3 tumor‐bearing mice and measured the uptake in the tumor by PET. The PET/CT images of a mice with a PC3 or LNCaP tumor on the right hind leg (Figure S2A) showed most pronounced [^18^F]FDG uptake in the outer part of the tumor and less in the center, suggestive of a necrotic center for the PC3 tumor and inhomogeneous uptake of [^18^F]FDG in the LNCaP tumor. The presence of necrotic tissue (regions without uptake inside the tumor) and the differences seen in shape of the tumors in the [^18^F]FDG images correspond to the differences in *T*_2_‐weighted MR images described below (Figure 4). [^18^F]FDG avid tissue in tumor ROIs was segmented using a threshold of 30% of the maximum voxel intensity in the tumor ROI (Figure S2B). After correction for radiotracer decay and body weight of the mouse, standard uptake values (SUVs) were calculated. Significantly lower SUV_max_ and SUV_mean_ values were found for LNCaP compared with PC3 tumors (*p* < 0.01) (Table 2 ).

**TABLE 2:** [^18^F]FDG PET estimated static (top) and dynamic (bottom) parameters

Static PET			
Tumor	SUV_max_	SUV_mean_	Biodistribution tumor/blood
PC3	1.61 ± 0.42	0.76 ± 0.16	4.12 ± 1.53
LNCaP	0.90 ± 0.18	0.49 ± 0.10	3.15 ± 0.75

Dynamic PET						
Tumor	*K*_i_ (Patlak)	*K*_i_ (2TCM 3K)	*V*_DND_ (2TCM 3K)	*K*_i_ (2TCM 4K)	*V*_D_ *(2TCM 4K)*	*V*_DND_ (2TCM 4K)
PC3	0.0075 ± 0.0025	0.0064 ± 0.0010	0.7318 ± 0.3706	0.0079 ± 0.0011	1.9055 ± 1.1040	0.6746 ± 0.3138
LNCaP	0.0049 ± 0.0037	0.0047 ± 0.0034	0.5003 ± 0.0823	0.0087 ± 0.0060	1.1045 ± 0.6555	0.4282 ± 0.1181
*t*‐test	*p* = 0.27	*p* = 0.41	*p* = 0.31	*p* = 0.82	*p* = 0.27	*p* = 0.21

Values for SUV_max_, SUV_mean_ and the ratio of tumor to blood ex vivo biodistribution are shown for PC3 tumors and LNCaP tumors in the top part. Dynamic parameters *K*_i_, *V*_DND_ and *V*_D_ are shown for both tumors with *p*‐values.

### **3.6** Kinetic modeling of PET data

To estimate the rates of glucose uptake and phosphorylation to glucose‐6‐phosphate, we measured the uptake of [^18^F]FDG dynamically in a subgroup of mice (PC3 *N* = 7, LNCaP *N* = 4). After reconstruction of the PET time frames and placing an additional ROI in the aorta to monitor the activity in blood over time, the mean decay‐corrected tumor absorption and blood activity‐time curves were fitted to 2TCMs. The different models resulted in different values for *K*_1_, *k*_2_, *k*_3_, *k*_4_ and consequently for [^18^F]FDG uptake rates *K*_i_ (=*K*_1_ *k*_3_/(*k*_2_ + *k*_3_)), volume of radiotracer distributions *V*_D_ (=*K*_1_ *k*_3_/*k*_2_ *k*_4_) and *V*_DND_ (=*K*_1_/*k*_2_). These values did not differ significantly for PC3 and LNCaP, while Patlak *K*_i_ values only showed a trend (Table 2).

### **3.7** Biodistribution

After killing the animals, tumor and blood were collected and ex vivo biodistributions were determined by measuring the radiotracer activity and calculating the uptake as percentage of initial dose per gram of tissue (%ID/g). The tumor/blood ratio was significantly higher (*p* < 0.05) for PC3 (4.12 ± 1.53) compared with LNCaP (3.15 ± 0.75) (Table 2).

### **3.8** Histopathological staining

To assess viable tumor tissue and necrosis or the presence of hemorrhages (presence of red blood cells), tumor slices were stained using H/E. Mice were excluded from the experiment if there was no viable tumor tissue present. This revealed that some of the LNCaP tumors contained (leaky) blood vessels or hemorrhages and several PC3 tumors had necrotic cores (lighter blue regions in the H/E stained coupes) (Figure 4). This is in line with the analyses of the *T*_2_‐weighted images and [^18^F]FDG uptake (vide supra). Percentage viable tissue varied between the histopathological slices and was 64% ± 23% for PC3 and 45% ± 26% for LNCaP tumors (*p* = 0.04).

### **3.9** Correlation between pyruvate to lactate exchange rate and FDG uptake

**FIGURE 6:**
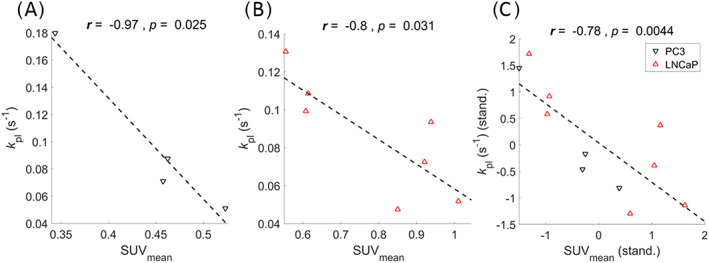
Correlation between dDNP and PET variables. Strong negative correlations are observed between rate constants *k*_pl_ (DNP) and SUV_mean_ (PET) values for LNCaP tumors (A) and PC3 tumors (B) separately, and standardized rate constants *k*_pl_ (DNP) and standardized SUV_mean_ (PET) values of both tumor groups combined (C)

To find a possible correlation between [1‐^13^C]pyruvate to lactate exchange and FDG uptake in our tumor models, we plotted the exchange rate constant *k*_pl_ and SUV as the [^18^F]FDG uptake parameter against each other and calculated Pearson correlations. A strong negative correlation was found between the values for SUV_mean_ and rate constant *k*_pl_ for both LNCaP (*r* = −0.97, *p* = 0.03, Figure 6A) and PC3 (*r* = −0.80, *p* = 0.03, Figure 6B). After standardizing both standard uptake values and rate constant *k*_pl_ for each tumor group, a strong negative correlation was also found between *k*_pl_ and SUV_mean_ for all tumors (*r* = −0.78, *p* < 0.01, Figure 6C). In addition to this, strong negative correlations were found between the pyruvate/lactate AUC ratios and the [^18^F]FDG uptake parameter *K*_i_ calculated from the Patlak plots of the PC3 tumors. A similar trend was observed for the correlation between *k*_pl_ and *K*_i_ for PC3 (Table 3, Figure S3).

**TABLE 3:** Bivariate Pearson correlations between DNP and PET values

	PC3 + LNCaP (standardized)	LNCaP	PC3	
	SUV_mean_ (*N* = 11)	SUV_mean_ (*N* = 4)	SUV_mean_ (*N* = 7)	*K*_i_ (Patlak) *(N =* 4)
*k*_pl_ (1/s)	*r* = −0.78 (<0.01)	*r* = −0.97 (0.03)	*r* = −0.80 (0.03)	*r* = −0.89 (0.11)
[lac/pyr]_fit_	*r* = −0.46 (0.15)	*r* = −0.94 (0.06)		*r* = −0.99 (0.01)
[lac/pyr]_raw_	*r* = −0.44 (0.18)	*r* = −0.97 (0.03)		*r* = −0.99 (0.01)

Overview of the strong negative correlations between rate constants *k*_pl_ (DNP) and lac/pyr ratios on one hand and multiple (PET) values SUV_mean_ and *K*_i_ (Patlak) on the other hand.

The ratio of *k*_pl_ and [lac/pyr]_fit_ and [lac/pyr]_raw_ over SUVs of [^18^F]FDG were calculated for the different SUVs, and significant differences between the two tumor types were found for some of the ratios (*p* < 0.05) (Table S2).

### **3.10** Conversion of hyperpolarized [1‐^13^C]pyruvate to lactate in prostate cancer cells

**FIGURE 7:**
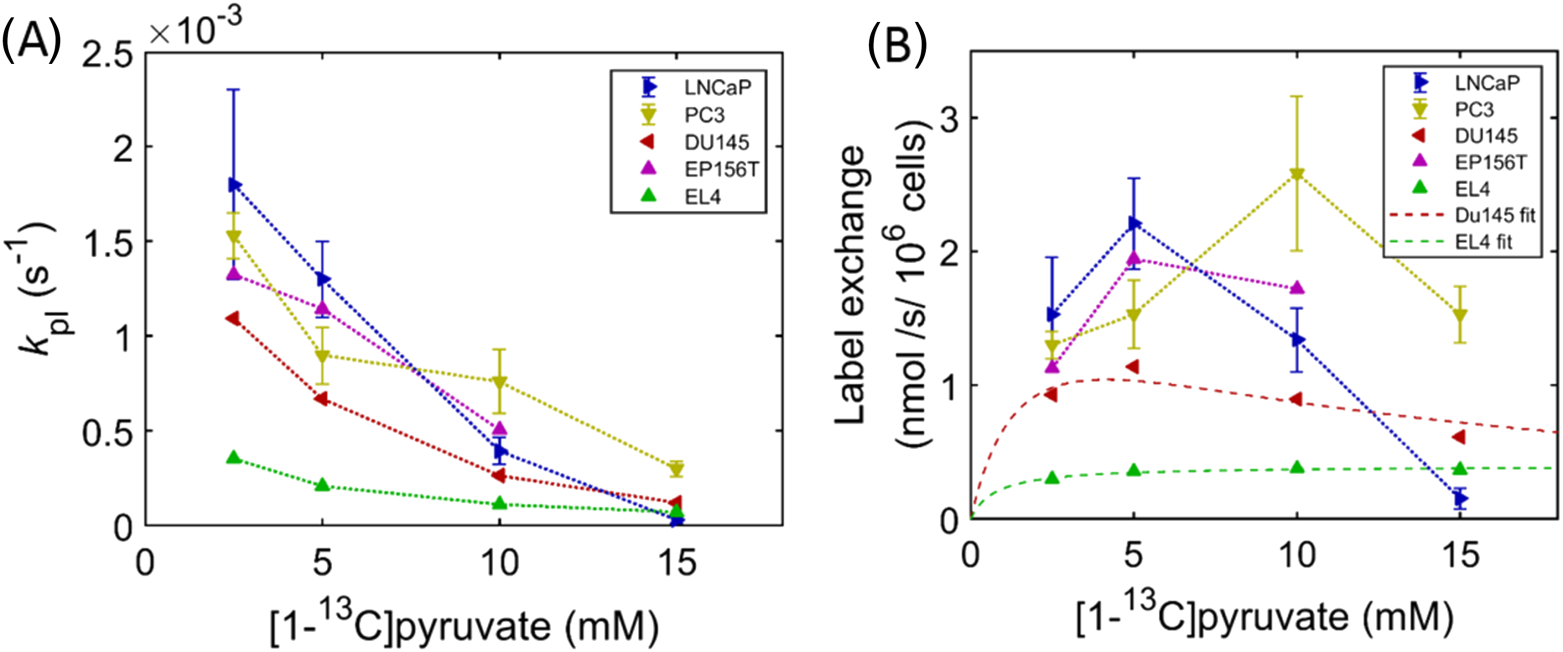
Pyruvate to lactate apparent exchange rate *k*_pl_ and exchange flux of LNCaP, PC3, DU145 and EP156T prostate cells in suspension and lymphoma EL4 cells as a function of the [1‐^13^C]pyruvate concentration. Apparent label exchange rates *k*_pl_ (A) and label exchange fluxes (B) are calculated and plotted against the supplied concentration of hyperpolarized [1‐^13^C]pyruvate. For human prostate cancer cell lines LNCaP, PC3 and DU145, an inhibition is seen at higher pyruvate concentrations. This is not seen for murine lymphoma cell line EL4. The flux data for EL4 and DU145 were fitted to Michaelis‐Menten equations (see text)

To better understand the results of the in vivo dDNP experiments on xenografts of PC3 and LNCaP tumors, we performed dDNP experiments on PC3 and LNCaP cell suspensions at different [1‐^13^C]pyruvate concentrations. The pyruvate to lactate exchange rate *k*_pl_ decreased with increasing pyruvate concentration from 2.5 mM to 15 mM for both cell lines; however, the pyruvate concentration dependences were different for the two cell lines (see Figure 7A). The average *k*_pl_ for 2.5 mM and 5 mM pyruvate was significantly higher for LNCaP ((1.80 ± 0.33) × 10^−3^/s for LNCaP and (1.22 ± 0. 13) × 10^−3^/s for PC3, *p* = 0.03), but was significantly lower for pyruvate concentrations of 10 mM and 15 mM ((0.212 ± 0. 07) × 10^−3^/s for LNCaP and (0. 52,985 ± 0. 11) × 10^−3^/s for PC3 respectively, *p* = 0.001). From these *k*_pl_ values we also calculated the label exchange flux. This flux increased for LNCaP cells from 2.5 mM to 5 mM and then decreased at higher [1‐^13^C]pyruvate concentrations. For PC3 cells this flux increased up to 10 mM pyruvate and then decreased at 15 mM (Figure 7B). For comparison we also investigated the effect of different pyruvate levels on the ^13^C exchange flux for human prostate EP156 cells, prostate cancer cells DU‐145 and lymphoma EL4 tumor cells. While EP156 and DU‐145 cells showed a similar pyruvate dependency as LNCaP cells (ie increasing up to 5 mM pyruvate and decreasing at higher levels), the exchange flux for EL4 cells steadily increased from 2.5 to 15 mM pyruvate (Figure 7B). The EL4 exchange flux values were fitted to a Michaelis‐Menten equation, which resulted in an apparent *K*_m_ of 0.74 mM and *V*_max_ of 0.4 nmol/s/10^6^ cells (Figure 7B). To take the apparent substrate inhibition into account we fitted the other curves to a modified Michaelis‐Menten equation: *V*_obs_ = *V*_max_[P]/(*K*_m_ + [P](1 + [P]/*K*_i_),^38^ in which we assumed substrate inhibition of LDH with an inhibition constant *K*_i_ of 9.4 mM.^28^ This was successful for flux data of the DU145 cells, resulting in an apparent *K*_m_ of 1.83 mM and *V*_max_ of 1.96 nmol/s/10^6^ cells (Figure 7B). For the other prostate cells a proper fit of the flux data to this equation was not possible, but it can be inferred from Figure 7B that the apparent *K*_m_ and *V*_max_ values will be higher.

## **4** DISCUSSION

In this study we monitored uptake and metabolism of glucose and pyruvate in tumor xenografts in mice of human prostate cancer cell lines LNCaP and PC3, representing early and late stage metastatic disease respectively, using [^18^F]FDG‐PET imaging and localized hyperpolarized [1‐^13^C]pyruvate MRS. The PET experiments revealed significantly higher SUVs for PC3 tumors compared with LNCaP tumors. However, the pyruvate to lactate ^13^C exchange rate constant *k*_pl_ was not different between the two tumor models. A significant strong negative correlation was found between the rate constants *k*_pl_ and [^18^F]FDG uptake values for both tumor groups and for the two tumor groups combined after standardization. Separate studies of LNCaP and PC3 cells in suspension revealed that the *k*_pl_ exchange rates of these cells are dependent on the pyruvate concentration, but in a different way for each cell type, which may be a factor determining *k*_pl_ values observed in vivo. Further studies, also including the human prostate tumor cell line DU145 and the immortalized prostate cell line EP156 in suspension, indicate inhibition of the pyruvate to lactate ^13^C flux in prostate cells by pyruvate at concentrations in the medium at about 5 mM and higher.

The significantly higher SUVs for the PC3 tumors compared with the LNCaP tumors agree with a more aggressive phenotype.^23^, ^25^, ^39^ The PC3 cells put a higher demand on glucose utilization, causing increased glucose uptake and consumption in comparison with LNCaP cells. The dynamic [^18^F]FDG uptake experiments showed higher values for the Patlak‐derived *K*_i_, and the parameters *V*_d_ and *V*_DND_ in PC3 tumors, which, although not significantly different, is consistent with the aforementioned significant increase in SUV. The variability in kinetic parameters between xenograft models has been shown to be much larger than the variability in standard uptake values,^39^ in agreement with our findings. This may be caused by the difficulty of reliably measuring an arterial input function from a small number of voxels, together with partial volume effects and spillover due to high uptake in surrounding tissue.

In contrast to [^18^F]FDG‐PET, the application of dDNP with [1‐^13^C]pyruvate allows to monitor metabolic conversions in tumors, most notably into ^13^C lactate. To quantify the conversion of [1‐^13^C]pyruvate to lactate we determined the ^13^C exchange rate constant *k*_pl_, by fitting the hyperpolarized pyruvate and lactate ^13^C signal time courses to a two‐site unidirectional kinetic model and also determined the lactate over pyruvate signal ratio. On the short time scale of the ^13^C MRS experiment a large part of the hyperpolarized pyruvate is still present in the blood.^40^ To take this into account, we included a PIF, *u*(*t*) = *u*_0_ exp{−*t*/*T*_bl_}, in the two‐site exchange model. Other, more complicated, PIFs have been proposed in the fitting procedure,^14^, ^33^ but using these resulted in poorer fits of our data with larger residuals compared with the simple PIF proposed here.

The *k*_pl_ values and lactate/pyruvate ratios were not significantly different between the two tumor models. These *k*_pl_ values are in the range of *k*_pl_ values that have been reported for subcutaneous tumors and relative standard deviations per tumor group are comparable.^33^, ^41^, ^42^, ^43^ In another model of prostate cancer, the TRAMP transgenic mouse, a significantly higher pyruvate‐to‐lactate *k*_pl_ was observed for tumors defined as high grade compared with those defined as low grade.^44^ A couple of factors may have influenced the *k*_pl_ values in our study. These could include variable contributions of muscle or necrotic tumor cells to the slice selected for data acquisition of tumor tissue. We minimized the contribution of muscle to this slice, and as we observed negligible label exchange between pyruvate and lactate in mouse leg muscle without tumor tissue we assume that *k*_pl_ values are not affected by the presence of muscle tissue. We also assume that necrotic tissue does not contribute to *k*_pl_ values, as it was recently demonstrated that necrotic tumor cells show no detectable pyruvate to lactate conversion.^45^

Other factors that may influence the ^13^C exchange rate constant *k*_pl_ are the rate of delivery of pyruvate to the tumor cells, the rate of transport of pyruvate over the membrane by monocarboxylate transporters (MCTs), the activity of LDH, the cellular steady‐state concentrations of NADH and NAD^+^ (nicotinamide adenine dinucleotide) as co‐factors in the LDH reaction, variable inhibition by substrates and products of transporter and LDH activity, and the concentration of lactate.^13^, ^46^, ^47^, ^48^

The presence of two pyruvate pools in the cytosol has been suggested in gliomas: one pool of glycolytically produced pyruvate in exchange with lactate and another derived from imported extracellular lactate, fueling the TCA cycle.^49^, ^50^, ^51^ The supplied pyruvate in our experiments could potentially end up in both pools, but the absence of a bicarbonate peak in our spectra and significant pyruvate to lactate conversion indicate that a second pyruvate pool involved in TCA cycle fueling can be neglected in our study.

Despite the increased sensitivity of the dDNP method it still requires rather high concentrations of pyruvate to be injected to obtain sufficient SNR, which may cause saturation effects that can critically affect metabolic parameters derived from dDNP experiments.^26^, ^27^ For murine lymphoma EL4 tumors in mice it was concluded that the rate of pyruvate delivery has little effect on the pyruvate to lactate exchange rate.^28^ A study involving the application of [1‐^13^C]pyruvate to rats showed decreased ^13^C exchange with lactate in heart, kidney and liver at applied pyruvate concentrations of more than 20 mM, which was ascribed to decreased activity of LDH.^26^ We observed that the ^13^C label exchange rate *k*_pl_ between pyruvate and lactate for the cancer cell lines LNCaP and PC3 in suspension depends differently on the concentration of pyruvate in the medium. This may also play a role in the *k*_pl_ values measured in vivo in prostate cancer xenograft models.

It is known that LDH can be inhibited by its substrate pyruvate at sufficiently high concentrations due to the formation of an abortive ternary complex with LDH and NAD^+^ and a binary complex with the adduct of NAD^+^ and pyruvate.^28^, ^52^, ^53^ To investigate if any exchange inhibition occurred for prostate cells we have determined exchange flux values for the human metastasis cancer cells LNCaP, PC3, DU145 and immortalized prostate cell line EP156 as a function of pyruvate levels in the medium and compared these with those for lymphoma cell line EL4. Remarkably, all the prostate cell lines showed a decrease in the flux rates above a medium concentration of 5 mM, indicating inhibition of the LDH reaction and/or cell membrane transport of pyruvate. This is in contrast to our measurement on EL4 cells, which showed no inhibition over this concentration range, in agreement with a previous similar study on this cell line^28^; Michaelis‐Menten analysis yielded comparable results in the two studies: apparent *K*_m_ was 0.74 and 0.88 mM respectively, and *V*_max_ was 0.40 and 0.68 nmol/s/10^6^ cells respectively. Another study on breast tumor cells TD47 also showed no inhibition of pyruvate, with an apparent *K*_m_ of 2.14 mM and *V*_max_ of about 0.45 nmol/s/10^6^ cells.^28^, ^46^ We fitted the exchange flux data of the prostate cells as a function of [1‐^13^C]pyruvate in the medium to a Michaelis‐Menten equation taking simple substrate inhibition into account with an inhibition constant of pyruvate as derived for rabbit muscle LDH.^28^ A good fit was possible for DU145 cells, but not for the other cells, indicating a more complex inhibitory effect of pyruvate. in vitro studies indicated that inhibition of LDH can occur at pyruvate concentrations above 1 mM, but that it strongly depends on the LDH concentration and the presence of other dehydrogenases competing for NAD^+^.^28^, ^54^ It has been reported that both LNCaP and PC3 cells have a higher LDH activity than non‐malignant PNT1A prostate cells, and that this activity is significantly higher in PC3 than in LNCaP cells.^55^ The higher *V*_max_ for [1‐^13^C]pyruvate to lactate exchange of the prostate cells relative to those of EL4 and T47D cells may also be due to a higher NAD(H) level in the prostate cells. A ^31^P NMR study of LNCaP cells indicated a high NAD level relative to nucleoside triphosphates.^56^ At the same time the inhibition of LDH activity may come from higher NAD, as this inhibition is caused by a ternary LDH‐NAD‐pyruvate complex and thus is determined by a combination of high NAD and pyruvate levels. It has also been concluded from metabolic control theory that the control of the ^13^C label exchange between pyruvate and lactate in EL4 cells is shared by membrane MCT and LDH activity. In particular, higher *K*_m_ values for the prostate cells would indicate that membrane transport contributes to the control of the ^13^C exchange.^28^ The expression of MCT4 is increased in LNCaP and PC3 relative to the non‐cancerous PNT1A cells.^57^

In our study the application of [1‐^13^C]pyruvate to the tumor‐bearing mice will result in an initial blood concentration of about 10 mM, only taking dilution into account, but this will subsequently decline due to uptake in various organs. Under similar experimental circumstances as in our study, the total pyruvate concentration in blood of mice 30 s after its application was estimated to be 3.0 ± 1.2 μmol/g,^58^ which corresponds to a concentration in plasma of about 5 mM. Thus, in the first 30 s of its application pyruvate in plasma will be in the range for which we observed differential *k*_pl_ variations between LNCaP and PC3, and ^13^C exchange inhibition. As its availability in the interstitial space next to the tumor cells may differ between PC3 and LNCaP the *k*_pl_ values may be affected by the local pyruvate levels.

Differences in vascularization might limit the uptake of pyruvate in parts of the tumors on the short time scale of the hyperpolarization experiments more than it influences the uptake of [^18^F]FDG on the much longer time scale of its observation. Although we have not quantified perfusion in our xenograft models, visual inspection of the *T*_2_‐weighted MR and [^18^F]FDG‐PET imaging and H/E stains indicated tissue heterogeneity that might have affected *k*_pl_ values.

In another study, on canine tumors, in which [^18^F]FDG‐PET and pyruvate‐to‐lactate exchange were combined across animals, neither SUV nor *k*_pl_ values could differentiate between carcinomas and sarcomas, although the ratio of *k*_pl_ over SUV_mean_ was significantly higher in sarcomas than in the carcinomas, which was taken as indicating a difference in contribution of the Warburg effect to energy metabolism in these tumors.^59^ The *k*_pl_/SUV ratio in our study was significantly higher in the LNCaP compared with the PC3 tumors.

Higher SUVs of [^18^F]FDG for tumors indicate higher glycolytic fluxes with more lactate production. Therefore, a positive correlation between SUV and *k*_pl_ exchange rate constant values for the xenograft tumors was expected. In contrast, we found a significant strong negative correlation between the 1‐^13^C pyruvate to lactate rate constants *k*_pl_ and SUVs for both tumor groups separately and combined. This suggests that glucose and/or pyruvate metabolism is different from that observed in other tumor types for which combined [^18^F]FDG‐PET and hyperpolarized [1‐^13^C]pyruvate MRS revealed a positive correlation between measures of pyruvate to lactate conversion and glucose uptake, or no correlation at all.^41^, ^59^, ^60^, ^61^ For example, in a subcutaneous rat breast cancer model, tumors with similar apparent diffusion coefficients (ADCs) showed a significant positive correlation between the label exchange rate *k*_pl_ between pyruvate and lactate and [^18^F]FDG standard uptake values.^41^ A (significant) weak negative correlation was found between ADC values and SUV_mean_ and a trend towards a weak negative correlation between ADC values and *k*_pl_. This suggests an effect of tumor cellularity on both measures of energy metabolism. A study in dogs showed a positive correlation between the label exchange rate between pyruvate and lactate and [^18^F]FDG uptake in sarcoma lesions, but not in carcinomas.^58,59^ In a subcutaneous hepatocellular carcinoma model, no correlation of pyruvate and lactate parameters with physiologic or histologic parameters was found.^62^ Studies comparing [^18^F]FDG‐PET and [1‐^13^C]pyruvate MR measurements before and after treatment showed an earlier decline in pyruvate to lactate exchange rate compared with decline in [^18^F]FDG uptake.^60^, ^61^ Inhibition of MCT1, the main pyruvate entry into the cell, slightly increased the SUV of [^18^F]FDG in mice with mammary fat pad xenograft tumors.^63^ All these findings indicate that the pyruvate to lactate production and glucose uptake as assessed by [^18^F]FDG‐PET are not necessarily correlated, and may differ between different tumor models and conditions of tumor growth. As the [1‐^13^C]pyruvate to lactate exchange rate *k*_pl_ can be affected by several parameters, it is difficult to explain the negative correlation with SUV_mean_ observed in our study. It could be that a higher uptake of glucose leads to higher pyruvate concentrations in the cell and that, together with the additional pyruvate provided in the hyperpolarization experiment, LDH activity is inhibited. Alternatively, higher glucose uptake may decrease MCT1 activity or vice versa, but this has not been described for prostate cancer cells yet. Although inhibition of MCT1 results in lower lactate/pyruvate AUC ratios in dDNP experiments on human‐derived cancer cells using [1‐^13^C]pyruvate,^64^ this does not necessarily mean that this transporter is rate limiting when it is operational. The SUV_mean_ and *k*_pl_ values we obtained are both metabolic measures representing the whole tumor. A comparison of spatially resolved detection of *k*_pl_ with spatially resolved SUVs might give more specific insight into their relationship.

A recognized problem with using the rate of pyruvate‐lactate exchange *k*_pl_ as an outcome measure in hyperpolarized [1‐^13^C]pyruvate experiments on tissues is the many factors that can influence this rate constant (vide supra). It is metabolically more meaningful to assess isotope flux, which requires us to know the local concentration of labeled pyruvate. For experimental EL4 murine lymphomas this was successfully determined using [1‐^14^C] labeling, but the need to use an additional modality further complicates dDNP experiments.^57,58^

In conclusion, we demonstrate a significant difference in glucose uptake by [^18^F]FDG‐PET between two xenograft models of human prostate cancer cells representing early and late stage metastatic disease, while no difference was observed for the apparent rate constant *k*_pl_ of ^13^C label exchange from pyruvate to lactate. This rate may be influenced by various factors; studies with the cells in suspension suggest that LDH inhibition by pyruvate may be one of these. A negative correlation was found between the two measures of tumor metabolism evaluated in this study, in contrast to the relation between these measures in other tumor models. This negative correlation indicates that [^18^F]FDG‐PET can be a useful complementary approach in dDNP studies of aggressive prostate cancer with [1‐^13^C]pyruvate.

## ABBREVIATIONS

[1‐^13^C]Pyr, (^13^C‐labeled) pyruvate; [^18^F]FDG, (^18^F‐labeled) fluorodeoxyglucose; [lac/pyr]_fit_/[lac/pyr]_raw_, ratio of pyruvate over lactate area under the curve values; ^13^C, carbon‐13; 2TCM, two‐tissue‐compartment model; ADC, apparent diffusion coefficient; AUC, area under the curve; CT, computed tomography; dDNP, dissolution dynamic nuclear polarization; FID, free induction decay; GLUT, glucose transporter; H/E, hematoxylin and eosin; IRW, Inveon Research Workplace; *k*_1_, rate of uptake of glucose from blood into the tissue; *k*_2_, rate of backflow of glucose from tissue into the blood; *k*_3_, rate of phosphorylation of fluorodeoxyglucose; *k*_4_, rate of dephosphorylation of fluorodeoxyglucose‐6‐phosphate; *K*_i_, influx rate constant of glucose; *K*_m_, Michaelis constant; *k*_pl_, exchange rate pyruvate‐lactate; Lac, lactate; LDH, lactate dehydrogenase; MCT, monocarboxylate transporter; PET, positron emission tomography; PIF, pyruvate input function; ppm, parts per million; PSA, prostate specific antigen; Pyr, pyruvate; ROI, region of interest; SNR, signal‐to‐noise ratio; SUV, (maximum or mean) standardized uptake value; *T*_1p_/*T*_1l_, *T*_1_ of pyruvate (p) or lactate (l); *t*_d_, time delay; *T*_E_, echo time; *T*_R_, repetition time; *V*_b_, fractional blood volume; *V*_D_/*V*_DND_, (non‐displaceable) distribution volume; *V*_max_, maximum reaction rate; *V*_obs_, observed reaction rate; *θ*, flip angle.

## FUNDING INFORMATION

Center for Medical Imaging North East Netherlands (PET MR project).

## Supporting information


**Figure S1.** **DNP time curve fits of pyruvate and lactate amplitudes fitted without including excitation flip angle *θ* as variable.** A LNCaP and B. PC3 tumors. *j*MRUI AMARES fitted amplitudes of pyruvate (red circles) and lactate (black triangles) with their corresponding curves fitted to the kinetic model in figure 3D, *k*_pl_, *u*_0_ and *T*_bl_ are fitted, *θ*=30°. Input function *u*(t) is shown in green. First point of the fit was the first point a pyruvate signal was detected.
**Figure S2. [** ^**18**^ **F]FDG PET images overlaid on CT images.** A. PET/CT overlay of a Balb/c nude mouse with PC3 tumor (top) and LNCaP tumor (bottom) on right hind leg of mouse. B. Tumor region in PET image of LNCaP tumor with ROI mask indicated. A 30%‐threshold was used to delineate [^18^F]FDG avid tumor issue. Images in A differently scaled than the image in B.
**Figure S3. Scatter plots of correlated DNP and dynamic PET variables.** Strong negative correlations between *lac/pyr* ratios (A, raio of fitted values, B, ration of raw data points) on one hand and *K*_i_ (PET) values for PC3 tumors. A similar trend is seen between *k*_pl_ and *K*_i_ (C). The number of LNCaP tumors measured with both DNP and dynamical PET is not enough to calculate this correlation since only a subset of tumors is measured using dynamical PET.
**Table S1. Injected [** ^**18**^ **F]FDG dose and tumor and mouse weights.** The injected dose of [^18^F]FDG per dataset is shown in MBq in the first column, the mouse weight and tumor weights are shown in the other columns per dataset.
**Table S2. Ratios of DNP values (*k***_pl_ **or *pyr/lac* ratios) over SUV values for PC3 and LNCaP.** Ratios of fitted rate constants *k*_pl_ (s^‐1^) and of AUC ratios of [*lac/pyr*] over SUV_max_ for LNCaP and PC3 tumors. Which fitting routine was used is indicated.
