# Supplementary material for: Pyruvate‐lactate exchange and glucose uptake in human prostate cancer cell models. A study in xenografts and suspensions by hyperpolarized [1‐13C]pyruvate MRS and [18F]FDG‐PET
Source: NMR Biomed. 2020 Jul 14;33(10):e4362. doi: 10.1002/nbm.4362 (PMC7507209; doi:10.1002/nbm.4362)
Supplement: Supplementary file 1 — Figure S1. DNP time curve fits of pyruvate and lactate amplitudes fitted without including excitation flip angle θ as variable. A LNCaP and B. PC3 tumors. jMRUI AMARES fitted amplitudes of pyruvate (red circles) and lactate (black triangles) with their corresponding curves fitted to the kinetic model in figure 3D, k pl, u 0 and T bl are fitted, θ=30°. Input function u(t) is shown in green. First point of the fit was the first point a pyruvate signal was detected. Figure S2. [ 18 F]FDG PET images overlaid on CT images. A. PET/CT overlay of a Balb/c nude mouse with PC3 tumor (top) and LNCaP tumor (bottom) on right hind leg of mouse. B. Tumor region in PET image of LNCaP tumor with ROI mask indicated. A 30%‐threshold was used to delineate [18F]FDG avid tumor issue. Images in A differently scaled than the image in B. Figure S3. Scatter plots of correlated DNP and dynamic PET variables. Strong negative correlations between lac/pyr ratios (A, raio of fitted values, B, ration of raw data points) on one hand and K i (PET) values for PC3 tumors. A similar trend is seen between k pl and K i (C). The number of LNCaP tumors measured with both DNP and dynamical PET is not enough to calculate this correlation since only a subset of tumors is measured using dynamical PET. Table S1. Injected [ 18 F]FDG dose and tumor and mouse weights. The injected dose of [18F]FDG per dataset is shown in MBq in the first column, the mouse weight and tumor weights are shown in the other columns per dataset. Table S2. Ratios of DNP values (k pl or pyr/lac ratios) over SUV values for PC3 and LNCaP. Ratios of fitted rate constants k pl (s‐1) and of AUC ratios of [lac/pyr] over SUVmax for LNCaP and PC3 tumors. Which fitting routine was used is indicated. [file NBM-33-e4362-s001.pdf]

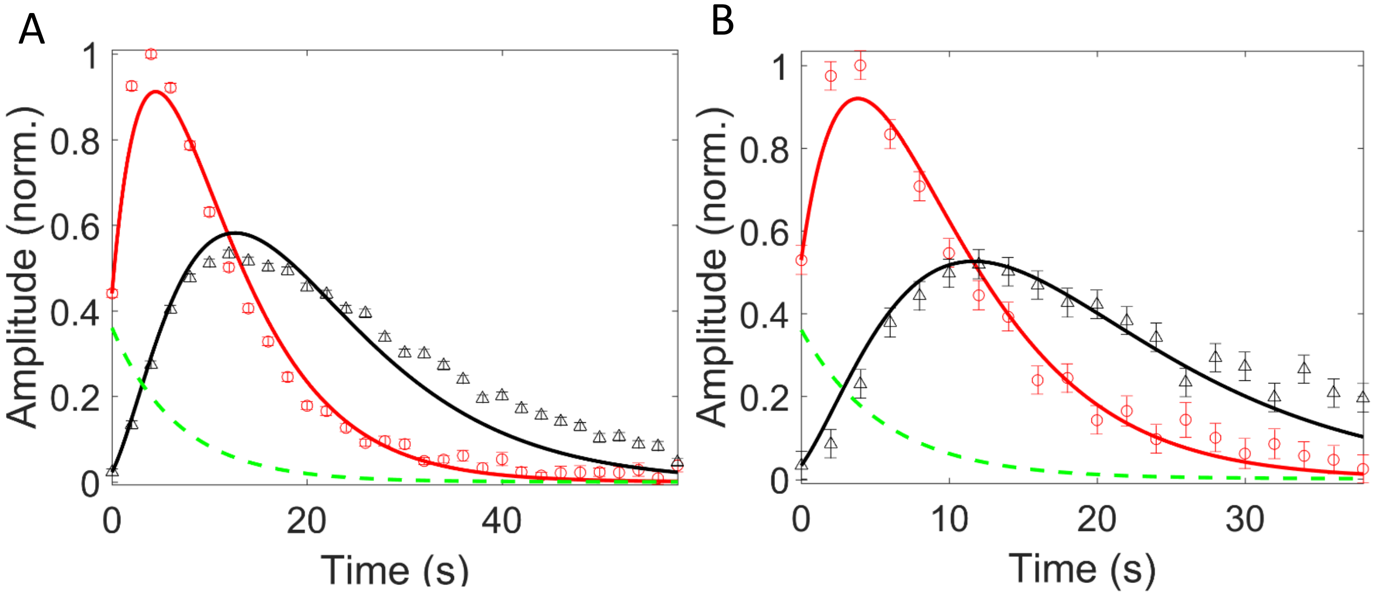

Figure S1. DNP time curve fits of pyruvate and lactate amplitudes fitted without including excitation flip angle θ as variable. A. LNCaP and B. PC3 tumors. *j*MRUI AMARES fitted amplitudes of pyruvate (red circles) and lactate (black triangles) with their corresponding curves fitted to the kinetic model in figure 3D, k_{pl}, u_0 and T_{bl} are fitted, θ=30°. Input function u(t) is shown in green. First point of the fit was the first point a pyruvate signal was detected.

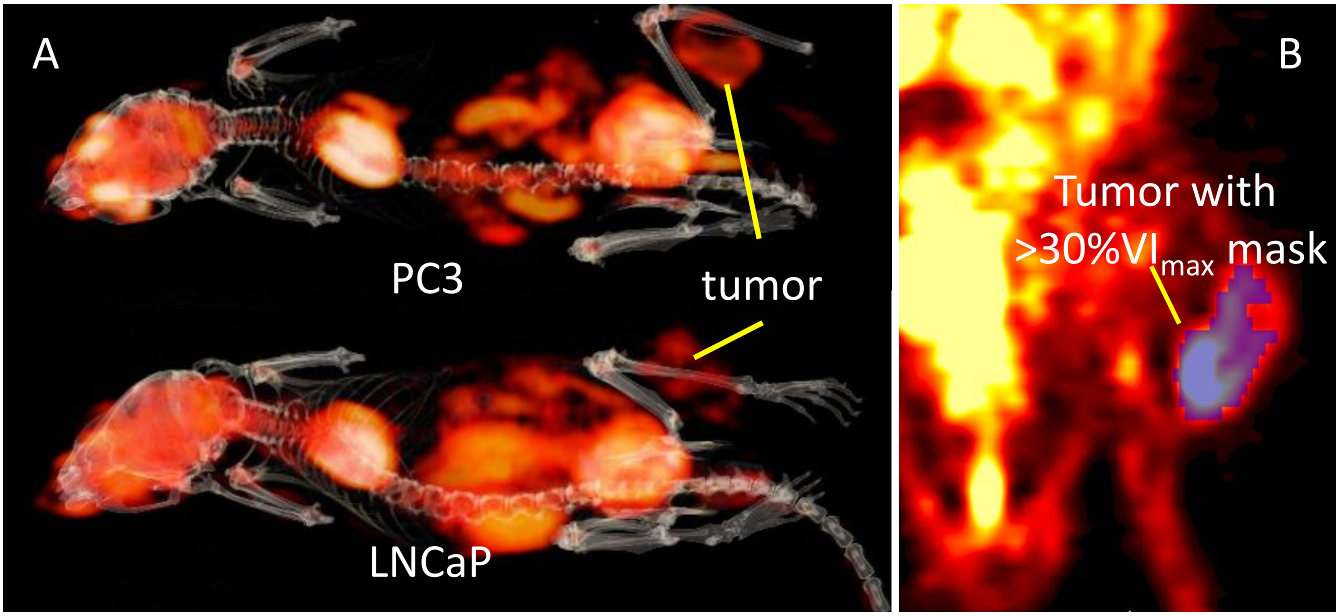

Figure S2. $[^{18}F]$FDG PET images overlaid on CT images. A. PET/CT overlay of a Balb/c nude mouse with PC3 tumor (top) and LNCaP tumor (bottom) on right hind leg of mouse. B. Tumor region in PET image of LNCaP tumor with ROI mask indicated. A 30%-threshold was used to delineate $[^{18}F]$FDG avid tumor tissue. Images in A are differently scaled than the image in B.

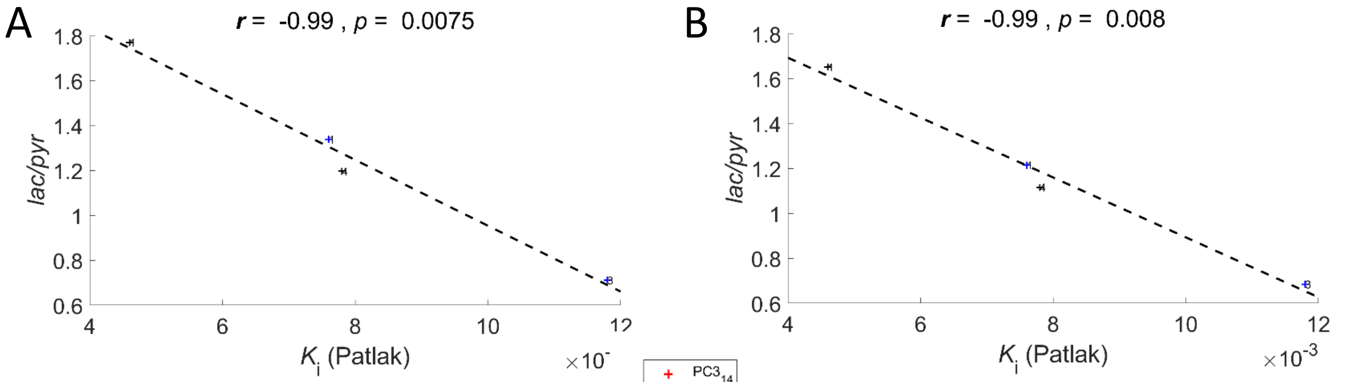

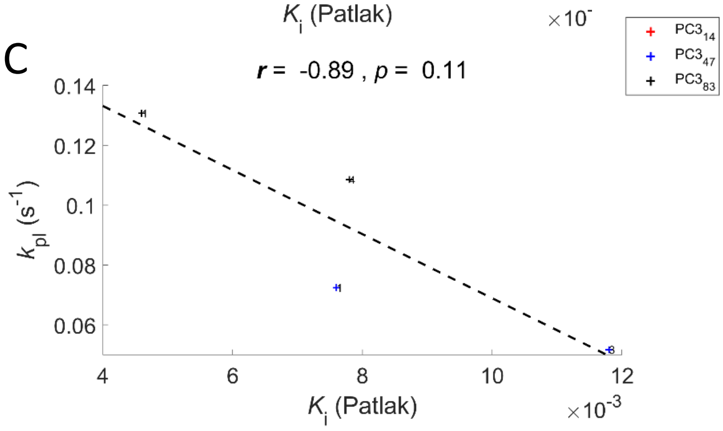

Figure S3. Scatter plots of correlated DNP and dynamic PET variables. Strong negative correlations between *lac/pyr* ratios (A, ratio of fitted values, B, ratio of raw data points) on one hand and and K_i (PET) values for PC3 tumors. A similar trend is seen between k_{pl} and K_i (C). The number of LNCaP tumors measured with both DNP and dynamical PET is not enough to calculate this correlation since only a subset of tumors is measured using dynamical PET.

Dataset	*Injected dose [^{18}F]FDG (MBq)*	*Mouse weight (g)*	*Tumor weight (g)*		
			All tumors	PC3	LNCaP
1	6.9 ±1.5	24.5 ±2.9	0.58 ±0.36	0.58 ±0.44	0.57 ±0.30
2	13.3 ±2.0	25.7 ±2.8	0.36 ±0.40	0.50 ±0.51	0.18 ±0.12
3	8.4 ±1.7	27.6 ±1.0	0.17 ±0.11	0.19 ±0.12	0.12 ±0.09

Table S1. Injected [^{18}F]FDG dose and tumor and mouse weights. The injected dose of [^{18}F]FDG per dataset is shown in MBq in the first column, the mouse weight and tumor weights are shown in the other columns per dataset.

**Parameters**	**Fitting routine used**	**Ratio LNCaP**	**Ratio PC3**	***p*-value**
k_{pl} / SUV_{max}	k_{pl}, u_0 T_{bl}, *start at* pyr_{max}	0.16 ±0.07	0.08 ±0.04	0.04
[*lac/pyr*]$_{fit}$ / SUV_{max}	k_{pl}, u_0 T_{bl}, *start at* pyr_{max}	1.77 ±0.88	0.82 ±0.40	0.03
[*lac/pyr*]$_{fit}$ / SUV_{max}	k_{pl}, u_0 T_{bl}, θ, *start at* pyr_0	1.54 ±0.73	0.77 ±0.40	0.05
[*lac/pyr*]$_{raw}$ / SUV_{max}	k_{pl}, u_0 T_{bl}, *start at* pyr_0	1.49 ±0.76	0.73 ±0.35	0.05
[*lac/pyr*]$_{raw}$ / SUV_{max}	k_{pl}, u_0 T_{bl}, *start at* pyr_{max}	1.71 ±0.79	0.82 ±0.38	0.03
[*lac/pyr*]$_{raw}$ / SUV_{max}	k_{pl}, u_0 T_{bl}, θ, *start at* pyr_0	1.49 ±0.76	0.73 ±0.35	0.05
[*lac/pyr*]$_{raw}$ / SUV_{max}	k_{pl}, u_0 T_{bl}, θ, *start at* pyr_{max}	1.71 ±0.79	0.82 ±0.38	0.03

Table S2. Ratios of DNP values (k_{pl} or *pyr/lac* ratios) over SUV values for PC3 and LNCaP. Ratios of fitted rate constants k_{pl} (s^{-1}) and of AUC ratios of [*lac/pyr*] over SUV_{max} for LNCaP and PC3 tumors. Which fitting routine was used is indicated.
